# From bulk, single-cell to spatial RNA sequencing

**DOI:** 10.1038/s41368-021-00146-0

**Published:** 2021-11-15

**Authors:** Xinmin Li, Cun-Yu Wang

**Affiliations:** 1 UCLA Technology Center for Genomics & Bioinformatics, Department of Pathology and Laboratory Medicine, David Geffen School of Medicine, UCLA, Los Angeles, CA USA; 2 Laboratory of Molecular Signaling, Division of Oral Biology and Medicine, School of Dentistry and Jonsson Comprehensive Cancer Center, UCLA, Los Angeles, CA USA; 3 Department of Bioengineering, Henry Samueli School of Engineering and Applied Science, UCLA, Los Angeles, CA USA

**Keywords:** RNA sequencing, Genome informatics

## Abstract

RNA sequencing (RNAseq) can reveal gene fusions, splicing variants, mutations/indels in addition to differential gene expression, thus providing a more complete genetic picture than DNA sequencing. This most widely used technology in genomics tool box has evolved from classic bulk RNA sequencing (RNAseq), popular single cell RNA sequencing (scRNAseq) to newly emerged spatial RNA sequencing (spRNAseq). Bulk RNAseq studies average global gene expression, scRNAseq investigates single cell RNA biology up to 20,000 individual cells simultaneously, while spRNAseq has ability to dissect RNA activities spatially, representing next generation of RNA sequencing. This article highlights these technologies, characteristic features and suitable applications in precision oncology.

## Introduction

Ribonucleic acid (RNA) has multiple forms and plays a critical role in cell growth and differentiation. RNA transcription and stability are tightly regulated in response to physiological and pathological stimuli^1^. Abnormal expression of RNA is frequently associated with human cancer initiation, development, progression and metastasis. In addition to the mutation of tumor suppressor genes and oncogenes, gene expression could be overactivated or epigenetically silenced which could lead to uncontrolled tumor cell growth and proliferation. Aberrant activation of cell growth signaling pathways and/or transcription factors could lead to high-level expression of genes associated with tumor development and progression. Different gene expression profiles may reflect different cancer subtypes, the stage of cancer development or tumor microenvironment^2–4^. Therefore, RNAseq is a powerful tool for understanding the molecular mechanisms of cancer development and developing novel strategies for cancer prevention and treatment.

## Bulk RNAseq

Since the first EST library was sequenced using Roche 454 sequencer in 2007^1^, bulk RNAseq has become the most valuable and extensively used tool in understanding cancer biology. Its diverse translational research and potential clinical applications have been well reviewed in the past^2–4^. This section does not intend to discuss either technologies or its applications in details. Instead, we will highlight the bottlenecks of its clinical translation and the recent progresses toward their solutions.

**Fig. 1:**
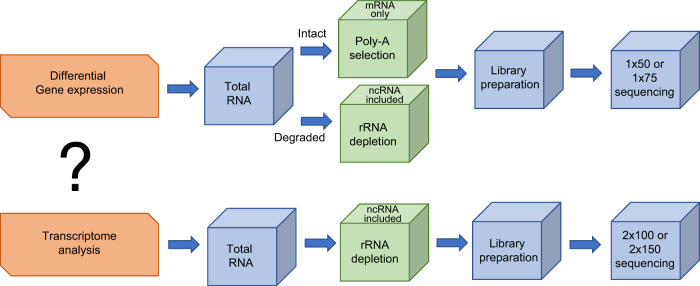
Outlines of two types of bulk RNAseq libraries. The types of libraries are dictated by the goal of the experiment: the single short read libraries are generally for differential gene expression, while the paired long read libraries are for whole transcriptome analysis, including spicing variant and point mutation analysis in addition to analysis of differentially expressed genes

By RNA species, bulk RNAseq involves sequencing two types of libraries: mRNA-only library and whole transcriptome library that includes all RNA species except for rRNA. By sequencing type, the most frequently used bulk RNAseq is a single end short sequencing focused on differentially expressed genes to understand molecular mechanisms implicated in various stage of tumorigenesis. This type of sequencing is simple and cost effective, largely focused on mRNA only. The less routinely used type is paired end longer sequencing aimed at additional knowledge on alternative splicing, point mutations, novel transcripts, long non-coding RNAs and gene fusions. This type of bulk RNAseq normally sequences rRNA-depleted libraries for more comprehensive information (Fig. 1).

Bulk RNAseq has broad utilities in cancer classification, biomarker and gene fusion discoveries, disease diagnosis, and optimizing therapeutic treatment. The translational research targeted for clinical oncology is primarily in two areas. The most informative area is the biomarker discoveries for cancer diagnosis, prognosis, and prediction. Numerous RNAseq-based signatures have been developed and validated across many major tumor types^5–9^. However, only a few such signature panels have been successfully translated into clinical practice due to low reproducibility. One of the main reasons for the poor performance across independent tumor patients is sampling bias inherent to intra-tumor heterogeneity^10^. A recent study provided a promising solution to tackle this issue through a novel strategy for biomarker selection^11^. By analyzing multiple RNAseq data from lung cancer patients, the team found that genes with homogeneous expression within individual tumors, despite high inter tumor variability, have significantly better prognostic potential. These genes “encode expression modules of cancer cell proliferation and are often driven by DNA copy-number gains”^11^. Such a transcriptomic signature minimizes sampling bias and offers robust prognostic performance in NSCLC survival. If this selection strategy is applicable in other tumor types, more biomarker signatures can be readily translated into clinical practice.

Another area subject to intensive clinical research is gene fusion discoveries. Fusion genes are well documented as major cancer drivers. Some recurrent fusion genes can be used as a diagnostic tool, such as the RUNX1–RUNX1T1 fusion for diagnosis of acute myeloid leukemia^12^, while others may be considered as prognostic biomarkers like TMPRSS2–ERG fusion in prostate cancer^13^. Using bulk RNAseq, numerous novel gene fusions have been discovered, many of which can be directly beneficial as FDA approved drugs or offer new therapeutic opportunities^14–17^. However, due to the high false positive rate and the low detection sensitivity, the diagnostic potential of these discoveries has not been fully realized in a clinical setting. Nevertheless, great advancements have recently been made towards addressing these two hurdles. A new computational modeling algorithm for fusion gene detection from bulk RNAseq data, named Data-Enriched Efficient PrEcise STatistical fusion detection (DEEPEST), was developed. DEEPEST is able to effectively minimize false positives and improve detection sensitivity^18^. In addition, Heyer EE et al^19^ reported a targeted RNAseq technique offering enhanced sensitivity and reduced background noise. Broad adoption of these new methodologies will facilitate accurate detection of gene fusion events for clinical applications.

One valuable potential of bulk RNAseq is still underappreciated. Because bulk RNAseq includes diverse types of RNA species and detects multiple forms of genomic alterations at a single base resolution, many of which are not detected by the DNA approach or other traditional methods^20–24^, it holds great promise to develop more efficient and multi-tasked clinical applications. FoundationOne Heme is such an excellent example. This clinical test detects various gene fusions, substitutions, indels, and CNVs, and used for therapy selection, prognosis, and diagnosis in multiple tumor types. The wide adoption of this LDT test in clinical labs has demonstrated that the multi-faceted, targeted RNAseq panel has a broad clinical utility in clinical oncology.

Bulk RNAseq uses a tissue or cell population as a starting material, and results in a mixture of different gene expression profiles from the studied material. The transcriptome programs of tumors are highly heterogeneous both between tumor cells, due to somatic genetic alterations, and within tumor microenvironments, resulting from significant infiltration of the stroma and other cell types in the tumor. The true signals driving the tumorigenesis or therapeutic resistance from a rare cell population or cell type can be obscured by an average gene expression profile from bulk RNASeq. This biological challenge catalyzed the birth of scRNAseq, an alternative RNAseq technology.

## Single cell RNA sequencing (scRNAseq)

Every tumor cell is unique with distinct somatic alterations, transcriptional regulations and epigenetic modifications. Differences between cells are even greater for RNA since all of these changes will be most likely reflected at the RNA level. Furthermore, RNA is more vulnerable to the influence of micro- and macro-environmental stimuli. Given the extraordinary transcriptional diversity at the single cell scale, tumor cells deserve and require individualized treatment at the transcriptome level. This demand facilitated the development of several scRNAseq technologies^25,26^. However, none of those have been broadly applied in translational or clinical research until the recent development of the 10X genomics chromium system, which triggered a rapid adoption of this revolutionized technology.

**Fig. 2:**
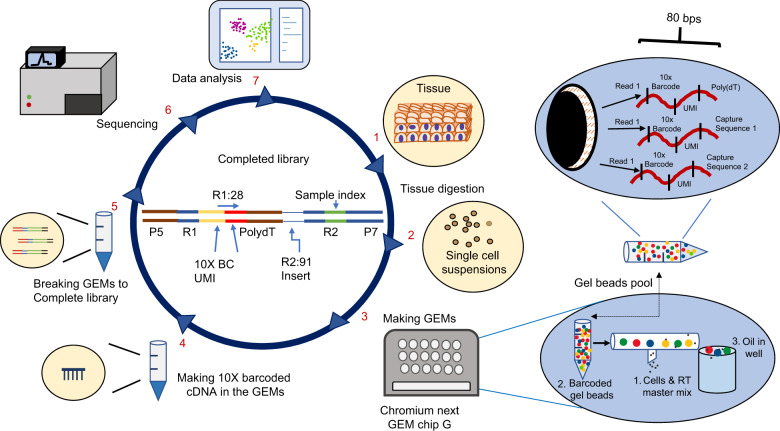
Workflow of 10X genomics single cell sequencing. The procedures include seven key steps. The library construction from steps 1 to 5 takes 2 days. Novaseq 100 cycle kits are normally used for sequencing. Sequencing sets up as read1:28 cycles for cell barcode & UMI, i7 index: 8 cycles for sample index, read 2: 91 cycles for insert (6). Cell Ranger, Loupe Browser and other commercial or publicly available software are used for data analysis (7)

10X genomics chromium system offers an integrated and complete solution for rapid analysis of gene expression profiles up to 20,000 individual cells in a single assay. The system consists of Chromium Controller or Chromium X for single cell partition, Chromium Connect for automated library construction, and several software package (cell ranger, loupe browser and cloud analysis) for data analysis. The core technology of the 10X single cell platform is the ability to generate hundreds of thousands of single cell microdroplets, called GEMs, on the Chromium microfluidics chip. Each GEM contains a single cell, reverse transcription mixes and a gel bead conjugated with millions of 80-base pair oligo sequences. Each oligo sequence includes an i7 adapter sequence for Illumina sequencing, a cell-specific 10x barcode for decoding the origin of the RNA, a random molecular tag for identifying and quantifying unique mRNA transcript (UMI), and an oligo-dT primer for mRNA binding. Each gel bead has a unique cell-specific 10x barcode, but all oligo sequences on the same bead contain an identical 10x barcode. There are 3.6 million different gel beads to ensure each of the GEMs has a unique 10x barcode. Thus, the microfluidics chip holds the key to make single cell GEMs while the cell-specific 10x barcode on the gel bead is the code to recognize and separate mRNAs from individual cells. Cell lysis commence immediately after cell encapsulation. The detailed workflow is outlined in Fig. 2.

Since 2017, scRNAseq has become one of the most popular genomic tools to dissect transcriptome heterogeneity, discovering rare cell types and cell states in tumors. Tumors are transcriptionally heterogeneous. These diversified transcriptional programs provide tumors with a plasticity to adapt to various environments^27^ and to promote tumorigenesis and treatment resistance^28^. This complex heterogeneity is partially due to the tumor cells themselves, derived from genetic variations among cells, as well as responses to other environmental factors. The tumor cell-derived heterogeneity is frequently morphologically indistinguishable. scRNAseq offers an unprecedented opportunity and has demonstrated its utility in dissecting intra-tumor heterogeneity at a single cell resolution in primary glioblastoma^29^, colorectal cancer^30^ and head and neck squamous cell carcinoma (HNSCC) or oral cancer^31^. Such a dissection is not only important for fundamental tumor biology, but is also effective for deciphering rare treatment-resistant cell populations and optimizing therapeutic strategy^32^. The partial epithelial-to-mesenchymal transition (p-EMT) has been identified to be associated with lymph node metastasis and adverse pathologic features. Tumor cells expressing the p-EMT program was found to be present at the invasive front of HNSCC^31^. Early scRNA-seq experiments found the presence of rare stem-like cells with treatment-resistance properties and a minor cell population expressing high levels of AXL that developed drug resistance after treatment with RAF or MEK inhibitors in melanoma^33^. Similarly, several drug-tolerant-specific RNA variants were also identified in a drug-tolerant breast cancer cell line that were absent in control cell lines^34^. These rare variants with significant implications are inaccessible by classic bulk RNAseq.

Tumor heterogeneity is also the result of the tumor microenvironment. Tumors are infiltrated by various immune and stromal cell populations that are constantly evolving and have been recruited from surrounding tissues. The diversity of different cell populations creates a unique tumor microenvironment. These non-tumor cells within the tumor also have distinct transcriptional programs and play important roles in tumor progression, metastasis, and cancer therapy resistance through constant communication with tumor cells. The characterization of transcriptional diversity of these non-tumor cells in the tumor is essential to the successful treatment of cancer patients. A recent scRNAseq study discovered that a high proportion of active CD8 + T lymphocytes are associated with a better outcome in non-small cell lung cancer^35^. A small subset of CD8 + T cells is associated with favorable response to adaptive cell transfer immunotherapy in melanoma patients^36^, while a large number of regulatory T lymphocytes have a poor prognosis in liver cancer^37^. Very recently, we showed that the immune checkpoint inhibitor targeting CD276 remodels the heterogeneity of HNSCC by reducing EMT and promote CD8 + T cell infiltration to kill cancer stem cells using scRNAseq^38^.

Another exciting application of scRNAseq is the identification of circulating tumor cells (CTCs) for non-invasive tumor diagnostics and treatment from a simple liquid biopsy. A recent report shows that a modified scRNAseq technique, Hydro-seq, has enhanced CTC capturing capacity while effectively minimizing background cells^39^. Using Hydro-seq technique, drug targets for hormone therapies were identified from CTCs of breast cancer patients. Although there are several CTC enrichment technologies available on the market, their clinical utility is limited by high background cells and lack of genetic knowledge of captured CTCs. Hydro-Seq provides a new approach for direct genetic characterization of CTCs at a single-cell resolution and opens a feasible path for making non-invasive tumor diagnosis, targeted therapy and treatment monitoring.

Until now, single cell sequencing has been centered around scRNAseq. However, scRNAseq data alone does not provide insight into upstream regulatory networks or downstream functional consequences. Integrated scRNAseq analysis with other single cell omic data will link these networks together and provides additional values in decoding complex causal relationships among different omic data. Recently, integrated single-cell multimodal omics is emerging as a major step toward interactively dissecting tumor heterogeneity at multiple layers of genetics. Nature Methods named single-cell multimodal omics as the method of the Year in 2019^40^ since it selected single cell sequencing as the method of the Year in 2013^41^. Several recent publications have thoroughly reviewed the utility of integrating scRNAseq data with other types of omic data^42,43^. It is highly expected that single-cell multimodal omics analysis will become a major focus in the upcoming years and reveals a more in-depth understanding of intratumoral heterogeneity of tumor cells and tumor microenvironment than scRNAseq alone.

scRNAseq, jointly with other genetic, epigenetic and proteomic data, will have a profound impact on cancer research. However, scRNAseq has several limitations, including: 1) the requirement for cell dissociation, removal of cell debris and dead cells to obtain viable, individual fresh cells, which can potentially stress the cells and alter the transcriptional profile; 2) the data only recover a few thousand unique transcripts from a single cell, far less than a whole transcriptome profile, that limits its broad applications; and 3) scRNAseq data (as well as bulk RNAseq data) loses critical spatial information, which negatively impacts the understanding of cell functionality and pathological changes^44^. Driven by these limitations, spRNAseq is emerging as a game changing technology.

## Spatial RNA sequencing (spRNASeq)

spRNAseq is a recently developed transformative technology. It combines the strengths of the global transcriptional analysis of bulk RNAseq and in situ hybridization, providing whole transcriptome data with spatial information. Tumors comprise of diverse cell types that often communicate in highly structured manners both spatially and temporally. Unlocking such complex spatial structures enables us to understand how tumor cells communicate with each other, escape immune surveillance, develop drug-resistance, and eventually metastasize. Thus, to fully understand tumorigenesis and design effective treatment strategies, it is essential to study gene expression spatially^45^.

**Fig. 3:**
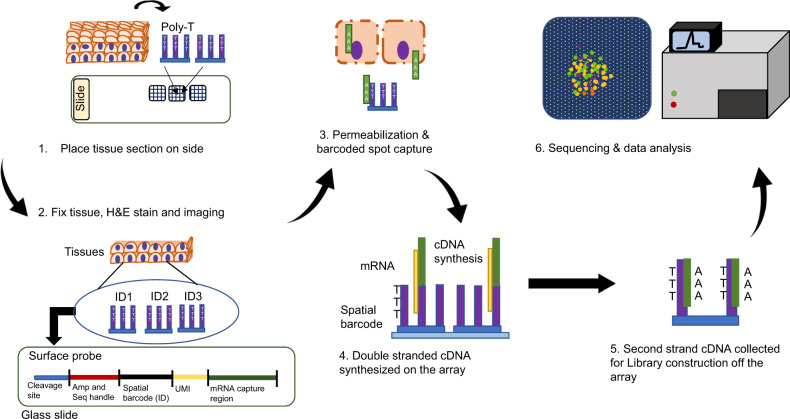
Outlines of 10X spatial transcriptomics. The workflow can be divided into two stages: on the array and off the array. The works on the array consist of sample preparation, staining & imaging, permeabilization and cDNA synthesis (1–4). The works off the array include library construction, sequencing & data analysis (5–6). Whole transcriptome libraries are sequenced using Novaseq V1.5 100 cycle kits (read 1: 28 cycles, i7 index: 10 cycles, i5 index: 10, read 2: 90 cycles). Data are analyzed and visualized using Space Ranger and Loupe Browser. Loupe Browser enables direct comparison of gene expression with histology data

Two spatial RNAseq technologies are commercially available today: the 10X spatial transcriptomics from 10X Genomics and digital spatial profiler from Nanostring Technologies. 10X spatial transcriptomics was initially developed by Spatial Transcriptomics and was further improved after it was merged with 10X Genomics in 2018. The first 10X visium spatial transcriptomics platform was launched in late 2019. This technology utilizes the power of classic microarrays and the modern barcoding technology of 10x Genomics for whole transcriptome analysis^43^. The workflow starts with imaging the fresh-frozen tissue section placed on the visium spatial gene expression slide. The visium slide is functionalized with printed oligo capture probes. The composition of the probes is similar to that of the oligo sequences coated on the gel beads as described in the scRNAseq section. After the tissue section is fixed, stained, and imaged, it is permeabilized to release RNA to bind to adjacent capture probes for on-slide cDNA synthesis. The double stranded cDNA, carrying the spatial barcode, is denatured, and the second strand cDNA is collected for off-slide library preparation (Fig. 3). This technology does not require specialized equipment except for traditional histology tools and is easily adoptable within existing lab infrastructures. The currently released visium platform is not able to offer single-cell resolution as it is limited by the spot size and spacing. However, the spatial resolution has now been experimentally improved by 1 400x^46^, and we expect a commercial product with single cell resolution to soon follow after.

**Fig. 4:**
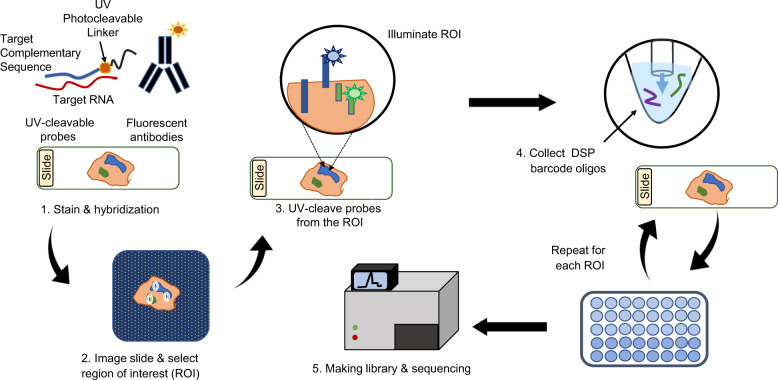
Workflow of GeoMx digital spatial profiler. The entire workflow takes 3 days. Day 1 for slide staining & hybridization, day 2 for slide processing on GeoMx DSP (2–4). During this phase, label cell types of interest with fluorescent morphology markers, load prepared slides onto the GeoMx DSP instrument, create fluorescent images, select regions of interest (ROIs), and then collect UV-cleaved oligos from these ROIs into the wells of a collection plate. Day 3 for library construction and sequencing (5). Sequencing sets up as read 1: 27 cycles, i7 index: 8 cycles, i5 index: 8, read 2: 27 cycles. The subsequent data analysis will use NanoString’s GeoMx NGS Pipeline software for converting FASTQ files to DCC digital count files, and the GeoMx DSP Data Analysis Suite for further analysis and visualization

The digital spatial profiler (DSP), GeoMx DSP, was launched in 2019. The GeoMx DSP is built on Nanostring’s digital molecular barcoding core technology and is further extended by linking the target complementary sequence probe to a unique DSP barcode through a UV cleavable linker. A pool of such barcode-labeled probes is hybridized to mRNA targets that are released from fresh or FFPE tissue sections mounted on a glass slide. The slide is also stained using fluorescent markers (i.e., fluorescently conjugated antibodies) and imaged to establish tissue “geography” using the GeoMx DSP instrument. After the regions-of-interest (ROIs) are selected, the DSP barcodes are released via UV exposure and collected from the ROIs on the tissue. These barcodes are sequenced through standard NGS procedures (Fig. 4). The identity and number of sequenced barcodes can be translated into specific mRNA molecules and their abundance, respectively, and then mapped to the tissue section based on their geographic location.

The DSP technology has several advantages over the 10X spatial transcriptomics technology, including profiling both RNA and protein, applying multiple samples on the same slide for cost savings, and flexibility to apply different protein or RNA panels for integrative analysis. The DSP whole transcriptome panel offers a targeted approach to quantify transcripts instead of the polyA approach like 10X transcriptome panel, thus excludes biologically uninformative high-expressors like ribosomal RNAs. It also allows user to add up to 60 custom targets of interest to quantify unique transcript variants such as isoforms, lncRNA and exogenous transcripts.

There are several common challenges in 10X visium and Nanostring GeoMx technologies that limit their applications, including non-single cell resolution, relatively low sensitivity, high cost, labor intensive process, and inability or limited capacity to do protein profiling. Both platforms cannot provide much needed single cell resolution today. The 10x Visium platform is limited by a spot size of 55 µm, which is larger than the size of many mammalian cells. Nanostring GeoMx can technically draw a region of interest at a single cell size, but high signal to noise ration limits this capacity. Before the resolution issue can be addressed, integration of scRNA-seq with spRNAseq is a viable option.

The spRNAseq is still in its early stages and has already shown promising applications that yielded inaccessible knowledge in biomarker discoveries, optimizing therapeutic strategy^47–49^, and in elucidating tumor heterogeneity and its dynamic microenvironments. Early spRNAseq studies, resulting from early product access programs, showed the coexistence of several distinct expression profiles from the same biopsy in melanoma that can be linked to specific histologic entities, but have more complex and detailed information than a histopathologic analysis can possibly reveal^50^. The lymphoid area adjacent to the tumor region displayed a specific expression pattern, which was later validated as a unique cancer expression profile that progresses gradually beyond tumor boundaries in prostate cancer^51^. Recent spRNAseq studies on tumor microenvironments revealed more insightful data. Using multimodal intersection analysis, Moncada R et al^52^ found that subpopulations of ductal cells, macrophages, dendritic cells and cancer cells have spatially restricted enrichments and coenrichments of inflammatory fibroblasts with cancer cells in primary pancreatic tumors. The distinct architecture among intercellular subpopulations and cross-talking networks within tumor microenvironments likely vary among patients and could have prognostic and therapeutic value. Similarly, Ji et al.^53^, identified four tumor subpopulations in cutaneous squamous cell carcinoma (cSCC). Of those, a tumor-specific keratinocyte (TSK) population unique to cancer is localized to a fibrovascular niche that is used as a hub for intercellular communication. In murine carcinomas, the co-existence of distinct subsets of cancer-associated fibroblasts (CAFs) has unique phenotypic features and functions that can be remodeled by TGFβ-blockade leading to enhanced efficacy of PD1 immunotherapy^54^. These findings demonstrate that the cell-cell interactions in a spatial context in the tumor microenvironment are critical components in understanding tumor progression, drug resistance and therapeutic efficacy.

## Concluding remarks

Three main RNAseq technologies described above have their unique features and suitable applications. It is likely that bulk RNAseq will remain the primary choice for clinical oncology in the near future. scRNAseq will further expand in the clinical research arena, and eventually get into the clinical practice when the cost significantly decreases. As for spRNASeq, it is still in its infancy. Given its capacity to dissect intercellular subpopulations of tumor microenvironment sensitively and spatially, spatial oncology will inevitably become a fundamental area of research in both discovery and therapeutics.

The ultimate goal in spatial biology is to develop a spatial multi-omics technology at a single cell resolution. Deng et al.^55,56^ has recently demonstrated the possibility of spatial genome wide epigenome profiling with three histone modification markers (H3K27me3, H3K4me3, H3K27ac) at tissue scale and cellular level. By combining epigenome, transcriptome and proteome datasets at a single cell resolution will have profound implications for understanding causative relationships of the multi-omics data, how genome organizes and functions, and how cancer develops.
